# The efficacy of parasacral transcutaneous electrical nerve stimulation for the treatment of overactive bladder in children: a systematic review and meta-analysis

**DOI:** 10.3389/fped.2025.1450634

**Published:** 2025-01-29

**Authors:** Zhuoqi Cheng, Yumeng Chai, Zhongbao Zhou, Yong Zhang

**Affiliations:** Department of Urology, Beijing Tiantan Hospital, Capital Medical University, Beijing, China

**Keywords:** child, overactive bladder syndrome, urinary incontinence, transcutaneous electric nerve stimulation, randomized controlled trials

## Abstract

**Aim:**

Despite the presence of published evidence in recent decades suggesting an improvement in overactive bladder (OAB) with the utilization of parasacral transcutaneous electrical nerve stimulation (PTENS), there is currently a lack of consensus guidelines for therapy. We conducted a meta-analysis to assess the impact of PTENS on children with OAB.

**Methods:**

A search was carried out using EMBASE, PubMed, and the Cochrane Controlled Register of Trials to find eligible randomized controlled trials (RCTs) published up to 1 May 2023. From the literature review, eight RCTs (351 participants) comparing PTENS and other treatments (standard urotherapy/anticholinergics/biofeedback/placebo stimulation) were considered.

**Results:**

The overall complete response rate with PTENS was 1.90 times that of children undergoing other treatment (relative risk 1.90, 95% confidence interval 1.45–2.49). No significant differences were observed in the mean dysfunctional voiding score system (*p* = 0.26), mean maximum voided volume (*p* = 0.79), average voided volume (*p* = 0.94), voiding frequency (*p* = 0.31), or reduction in the number of children with incontinence episodes (*p* = 0.81). However, regarding the reduction of children with constipation, the PTENS group demonstrated a better effect compared with the control groups (*p* = 0.01).

**Conclusions:**

In summary, PTENS has demonstrated better response rates and fewer side effects compared to conventional first-line treatments, such as standard urotherapy and antimuscarinic drugs. Clinicians should consider individual circumstances when treating children with OAB. However, it is important to note that the findings of this study are limited by the small sample size and imperfect outcomes. Further high-quality RCTs are needed to establish the most effective treatment protocol.

## 1 Introduction

The International Children's Continence Society (ICCS) (1) defines overactive bladder (OAB) as a condition characterized by urinary urgency, with or without urinary incontinence (UI), often accompanied by increased voiding frequency (VF) and nocturia, in the absence of urinary tract infection (UTI) or other evident organic pathology. A cross-sectional study (2) revealed that 21.7% of lower urinary tract symptoms (LUTS) in the Netherlands populations included stress incontinence, bedwetting, and urge incontinence. Among these, urge incontinence was reported in 5.8% of cases, which is more realistic. In addition, girls experience UI slightly more frequently than boys (2, 3).

The primary management approach for children with OAB involves standard urotherapy, encompassing education on normal lower urinary tract anatomy and function, behavioral modifications (establishing regular voiding habits, adopting proper voiding posture, avoiding holding maneuvers, etc.), lifestyle guidance (maintaining a balanced fluid intake and diet, reducing caffeine consumption, adhering to regular bladder and bowel emptying schedules, etc.), and monitoring symptoms and voiding habits (4). Pharmacological treatment may be considered for children who show a poor response to standard urotherapy and do not have voiding dysfunction. For medically refractory cases of OAB in children, specific urotherapies may be applied, including intermittent catheterization, electrical stimulation [such as parasacral transcutaneous electrical nerve stimulation (PTENS), tibial peripheral nerve stimulation, sacral neurostimulation], biofeedback, and pelvic floor muscle relaxation, among others (5). Approximately 20 years ago, Hoebeke et al. (6) first applied PTENS to children with OAB and UI, achieving a definitive cure rate of 51.2%. Subsequent studies reported varying rates of complete resolution with this management, with a range of 0%–70% (7, 8).

A previous meta-analysis by O’Sullivan et al. (9) was limited to one assessment indicator due to a small sample size. The purpose of this meta-analysis was to comprehensively evaluate the treatment effect of PTENS in pediatric patients with OAB. In addition, given the variation in assessment criteria and the absence of a standardized therapeutic protocol, this analysis aims to provide guidance for establishing a standardized protocol for PTENS in the future.

## 2 Materials and methods

### 2.1 Data sources and searches

**Figure 1:**
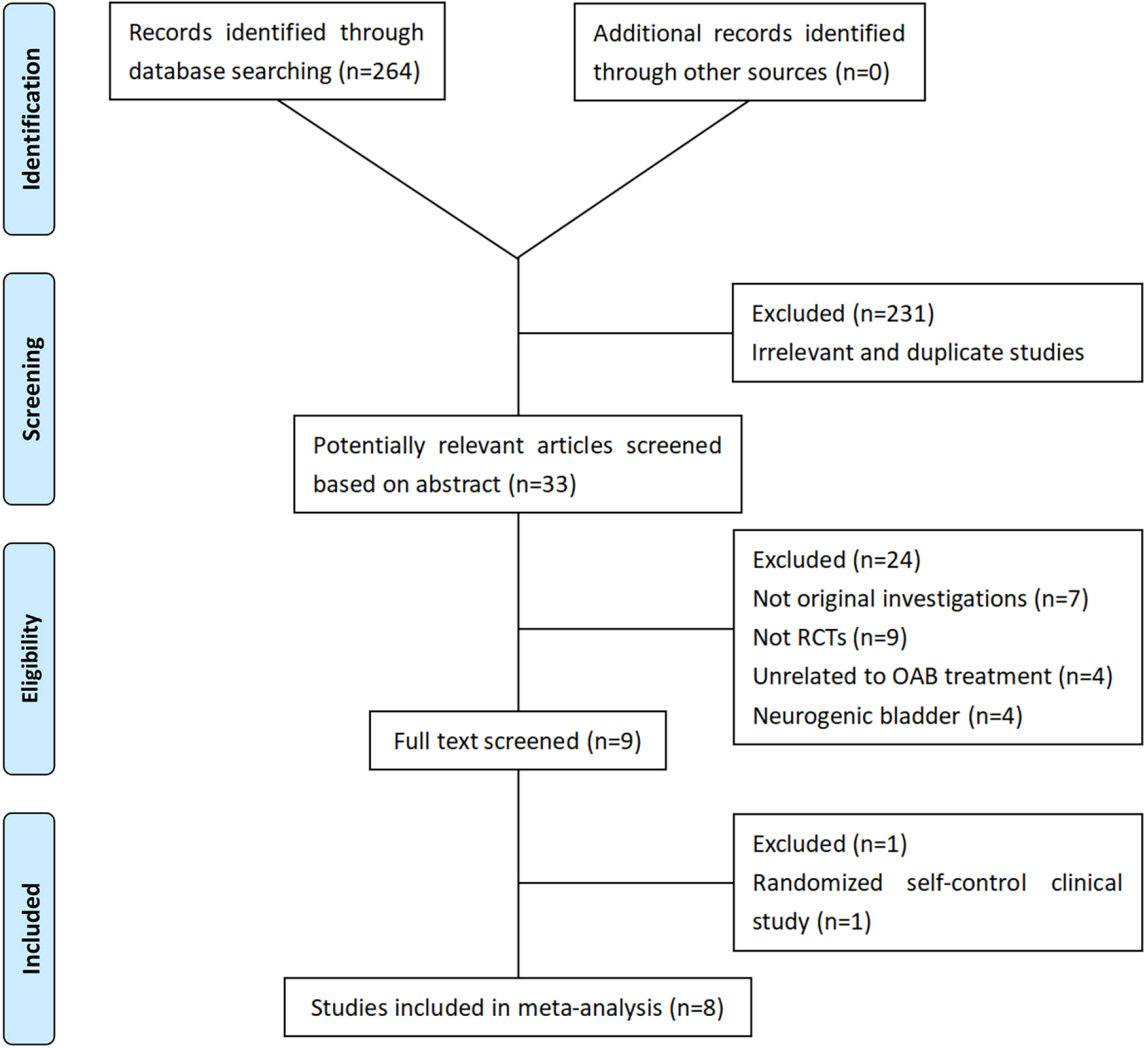
Flowchart of the study selection process. RCT, randomized controlled trials; OAB, overactive bladder.

To assess the efficacy of various strategies for pediatric OAB, we conducted a search using EMBASE, PubMed, and the Cochrane Controlled Register of Trials (CENTRAL) to identify eligible randomized controlled trials (RCTs) published up to 1 May 2023. The search terms included “transcutaneous electrical nerve stimulation OR TENS OR parasacral transcutaneous electrical nerve stimulation OR PTENS” and “overactive bladder OR OAB” and “pediatric OR children” and “RCTs.” In addition, we reviewed the references of relevant articles. The search strategy is outlined in Figure 1.

### 2.2 Inclusion criteria

**Figure 2:**
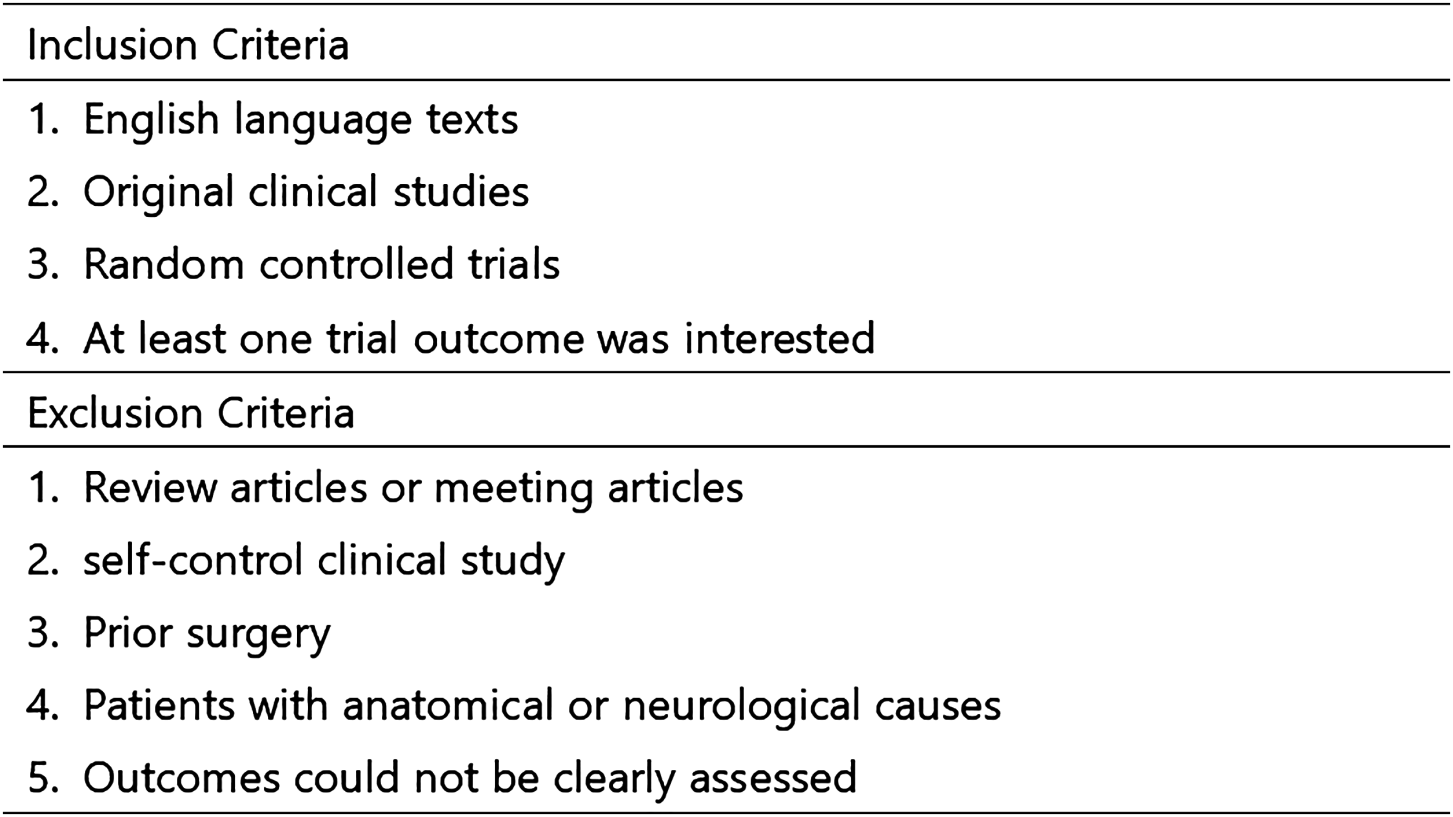
Inclusion and exclusion criteria.

Two independent reviewers (ZC and YC) conducted blind evaluations of the collected research studies to determine their suitability for inclusion in the ultimate meta-analysis. Studies were included or excluded based on assessments of their titles, abstracts, and complete papers, as outlined in Figure 2. Eight studies were ultimately included in the final meta-analysis (10–17).

### 2.3 Quality assessment

We evaluated the validity of the searched studies through a qualitative appraisal of their study designs and methods. We used the risk-of-bias tool recommended by the Cochrane Collaboration (18) for detailed quality assessment.

### 2.4 Data extraction

The following research information was extracted from the included studies: publication time, name of first author, therapy, sample size in different intervention groups, number of children who achieved a complete response to the treatment, mean dysfunctional voiding score system (DVSS), mean maximum voided volume (MVV), average voided volume (AVV), mean VF, number of children with incontinence episodes, and the reduction in the number of children with constipation. To compare PTENS treatment against controls, the primary outcome was the number of children who achieved a complete response. The results were dichotomized into two categories: complete response and partial or no response, as illustrated in Table 1. Then, a meta-analysis was performed.

**Table 1:** Results of PTENS versus controls.

	PTENS			Control		
	Complete response	Partial	No	Complete response	Partial	No
Casal-Beloy et al. (10)	24	6	10	11	8	27
de Abreu et al. (17)	8	10	2	7	12	1
de Paula et al. (16)	5		2	2		5
dos Reis et al. (15)	20	10	3	17	13	1
Hagstroem et al. (14)		8	5	1	1	10
Lordêlo et al. (13)	13	6				16
Quintiliano et al. (12)	6		7	3		10
Sillén et al. (11)	16	3	5	13	8	7

### 2.5 Statistical analysis

Data were analyzed using RevMan v5.3.0 (Cochrane Collaboration, Oxford, UK) (19). We summarized the complete response and the changes in mean number of DVSS, mean number of MVVs per 24 h, and mean number of AVVs per 24 h between baseline and the study endpoint. The mean difference (MD) was used to evaluate continuous data, while the risk ratio (RR) was employed to assess dichotomous data. Before conducting the meta-analysis, data that were not in the form of mean and standard deviation were accordingly (20). We analyzed comparable data using 95% confidence intervals (CIs). An individual study that could be characterized as a random-effects model was chosen. Under the random-effects model, the differences of the true effect sizes were allowed.

## 3 Results

A total of 264 studies were identified using the search terms outlined above. Eight RCTs were included in the final meta-analysis to analyze PTENS and other therapies for children with OAB. The basic information and patient demographics of these eight studies are summarized in Table 2, while the treatment characteristics are outlined in Table 3.

**Table 2:** Patient demographics and pre-treatment details of patients enrolled in PTENS RCTs.

Study	Country	Study design	Age/years		Sex (M/F)		Intervention group 1 (sample size)	Intervention group 2 (sample size)	Pre-treatment
			PTENS	Control	PTENS	Control			
Casal-Beloy et al. (10)	Spain	RCT	9 (7–12)	8 (7–15.5)	14/26	21/25	PTENS (40)	Oxybutynin (46)	Urotherapy
de Abreu et al. (17)	Brazil	RCT					Standard urotherapy + PTENS (20)	Standard urotherapy + sham electrotherapy (20)	No
de Paula et al. (16)	Brazil	RCT					PTENS (8)	Standard urotherapy (8)	No/at least 6 months free
dos Reis et al. (15)	Brazil	RCT	9.58 ± 2.89	9.19 ± 2.41	11/22	10/21	Standard urotherapy + PTENS (33)	Standard urotherapy + Biofeedback (31)	TENS (*n* = 2), Biofeedback (*n* = 2), Pharmacological (*n* = 11)
Hagstroem et al. (14)	Denmark	RCT	8.7 ± 2.0	8.5 ± 1.2	7/6	3/9	PTENS (13)	Placebo stimulation (12)	Urotherapy + Anticholinergic
Lordêlo et al. (13)	Brazil	RCT	7.5 ± 3	7.4 ± 2.8	8/13	4/12	Standard urotherapy + PTENS (21)	Standard urotherapy + superficial scapular electrical stimulation (16)	Urotherapy
Quintiliano et al. (12)	Brazil	RCT	6.3 ± 2.4	6.5 ± 2.0	4/9	5/10	Standard urotherapy + PTENS (13)	Standard urotherapy + Oxybutynin (15)	N/A
Sillén et al. (11)	Sweden	RCT	8 ± 1.9	8 ± 1.5	16/16	19/11	Standard urotherapy + PTENS (26)	Standard urotherapy (29)	Urotherapy (*n* = 3), Urotherapy + anticholinergic (*n* = 5)

PTENS, parasacral transcutaneous electrical nerve stimulation; RCTs, randomized controlled trials.

**Table 3:** Treatment details of PTENS in RCTs.

Study	Current intensity	Stimulation frequency (Hz)	Impulse (μs)	Periodicity	Treatment duration
Casal-Beloy et al. (10)	Threshold	10	200	20 min daily	6 months
de Abreu et al. (17)	Threshold	10	700	20 min × 3/week	6.5 weeks
de Paula et al. (16)	Threshold	10	700	20 min × 1/week	20 weeks
dos Reis et al. (15)	Threshold	10	700	20 min × 2/week	6 months
Hagstroem et al. (14)	Threshold	10	200	2 h daily	4 weeks
Lordêlo et al. (13)	Threshold	10	700	20 min × 3/week	6.5 weeks
Quintiliano et al. (12)	Threshold	10	700	20 min × 3/week	6.5 weeks
Sillén et al. (11)	Threshold	10	N/A	20 min × 2/day	12 weeks

PTENS, parasacral transcutaneous electrical nerve stimulation; RCTs, randomized controlled trials.

**Figure 3:**
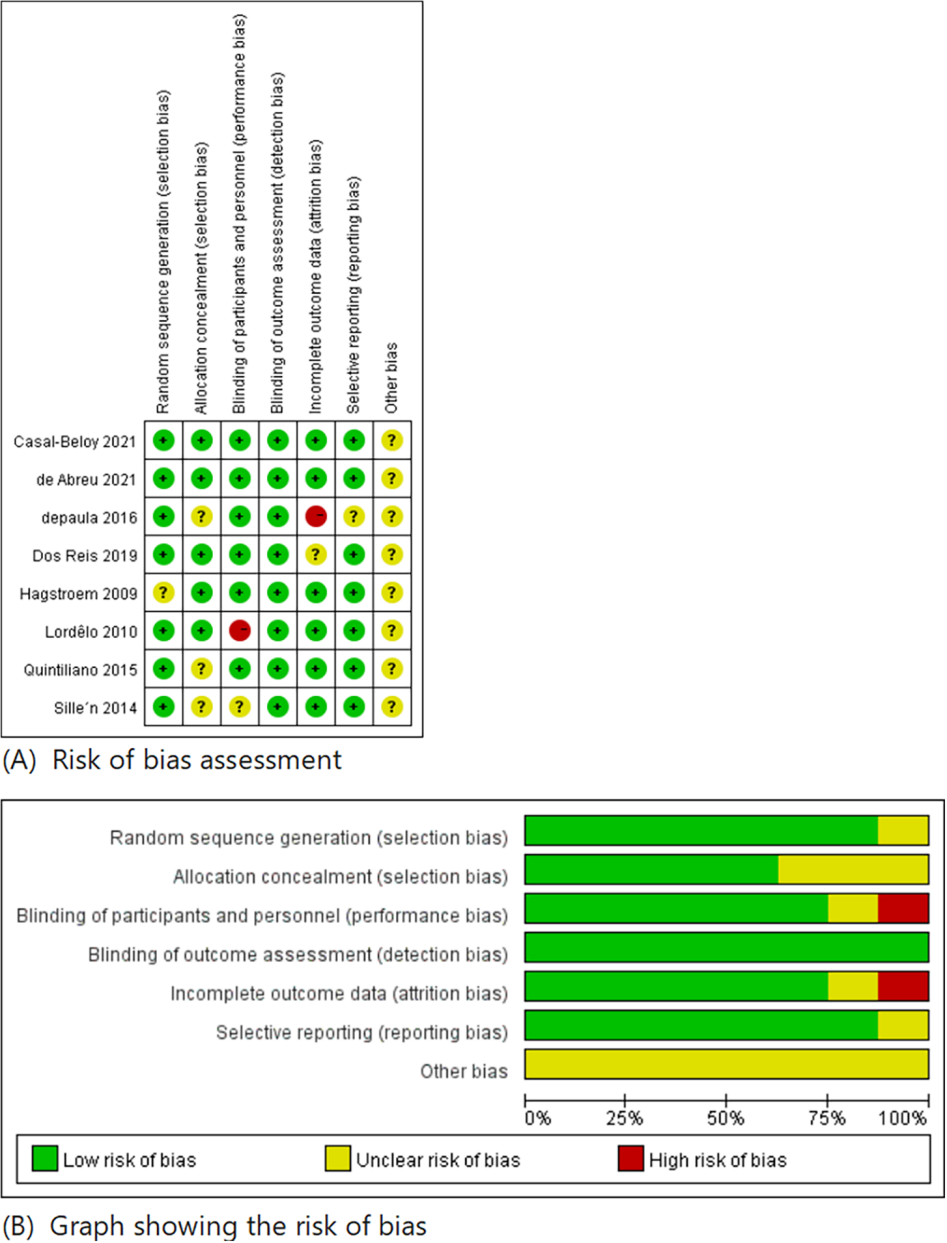
Risk of bias summary.

**Figure 4:**
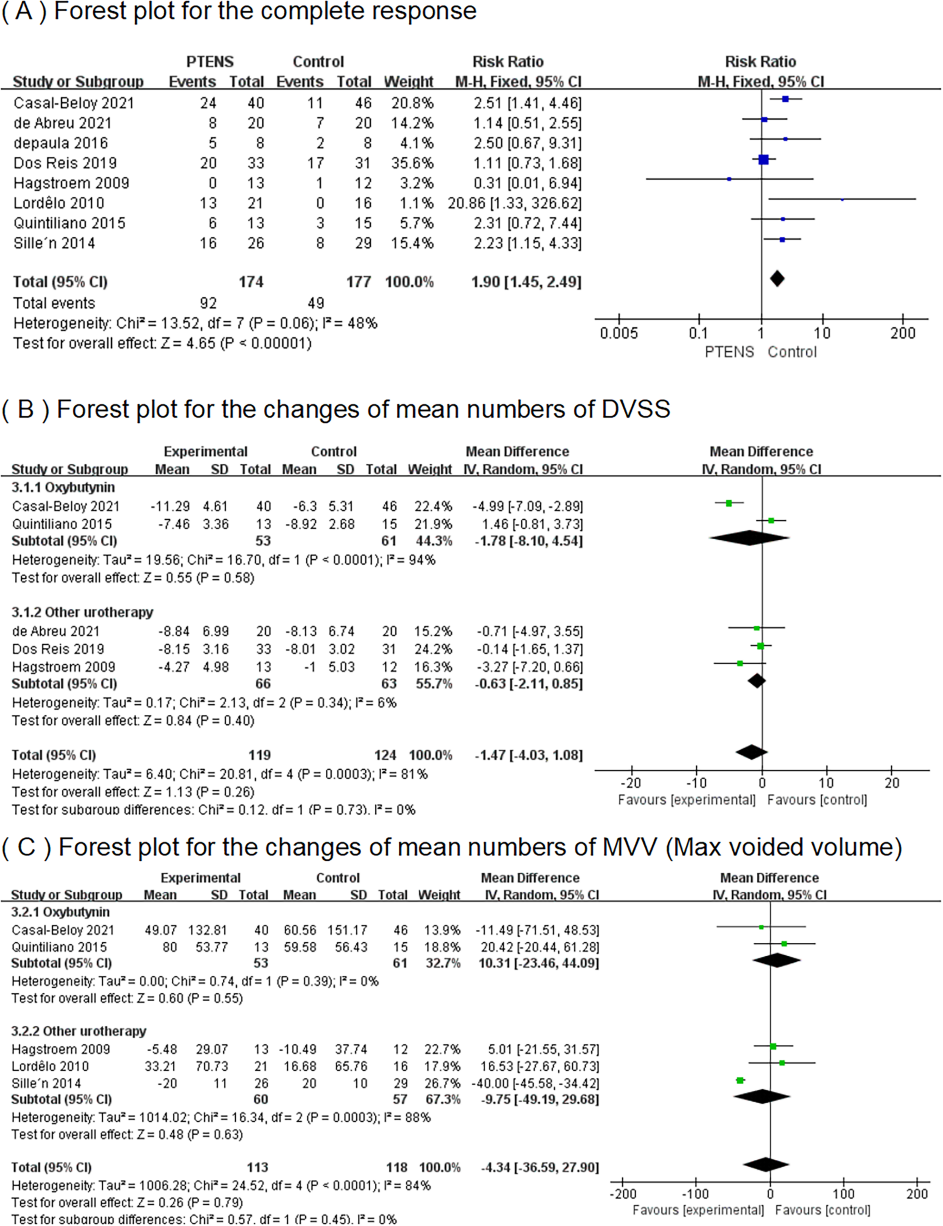
Results of complete response, DVSS, and MVV between the PTENS group and the control group.

All eight studies were RCTs, with each study detailing its randomization process. They were assessed using the methods outlined above, and the risk of bias of each study was presented in Figures 3 and 4.

### 3.1 The complete response

In eight RCTs (10–17), a total of 351 patients participated (174 in the PTENS group and 177 in the control group) in the analysis. The 95% CI was 1.15–4.33, with a RR of 1.90 (*p* = 0.06), indicating that the overall complete response rate with PTENS was 1.90 times higher than that of children undergoing other treatments (Figure 4).

### 3.2 The changes in mean numbers of DVSS

In five RCTs (10, 12, 14, 15, 17), 243 patients (119 patients in the PTENS group and 124 in the control group) had pre- and post-treatment data on the mean number of DVSS. A random-effects model showed the MD for the total was −1.47% (95% CI −4.03 to 1.08); the MD for oxybutynin was −1.78 (95% CI−8.10 to 4.54); and the MD for other urotherapy was −0.63 (95% CI −2.11 to 0.85). The forest plot demonstrated there were no significant differences between the PTENS group and the control group in the change in the mean number of pre- and post-treatment DVSS, regardless of whether the comparison was made within the oxybutynin subgroup or the other urotherapy group (Figure 4).

### 3.3 The changes in mean numbers of MVV

Five RCTs (10–12, 14, 17) including 231 patients (113 in the PTENS group and 118 in the control group) were included in this analysis. Comparing the patients receiving oxybutynin treatment and other urotherapy separately, the MVV in the PTENS group showed no significant difference (MD 10.31, 95% CI −23.46 to 44.09; MD −9.75, 95% CI −49.19 to 29.68). There were no statistically significant differences between the PTENS group and the control group (MD −4.34, 95% CI 36.59 to 27.90) (Figure 4).

### 3.4 The changes in mean numbers of AVV

**Figure 5:**
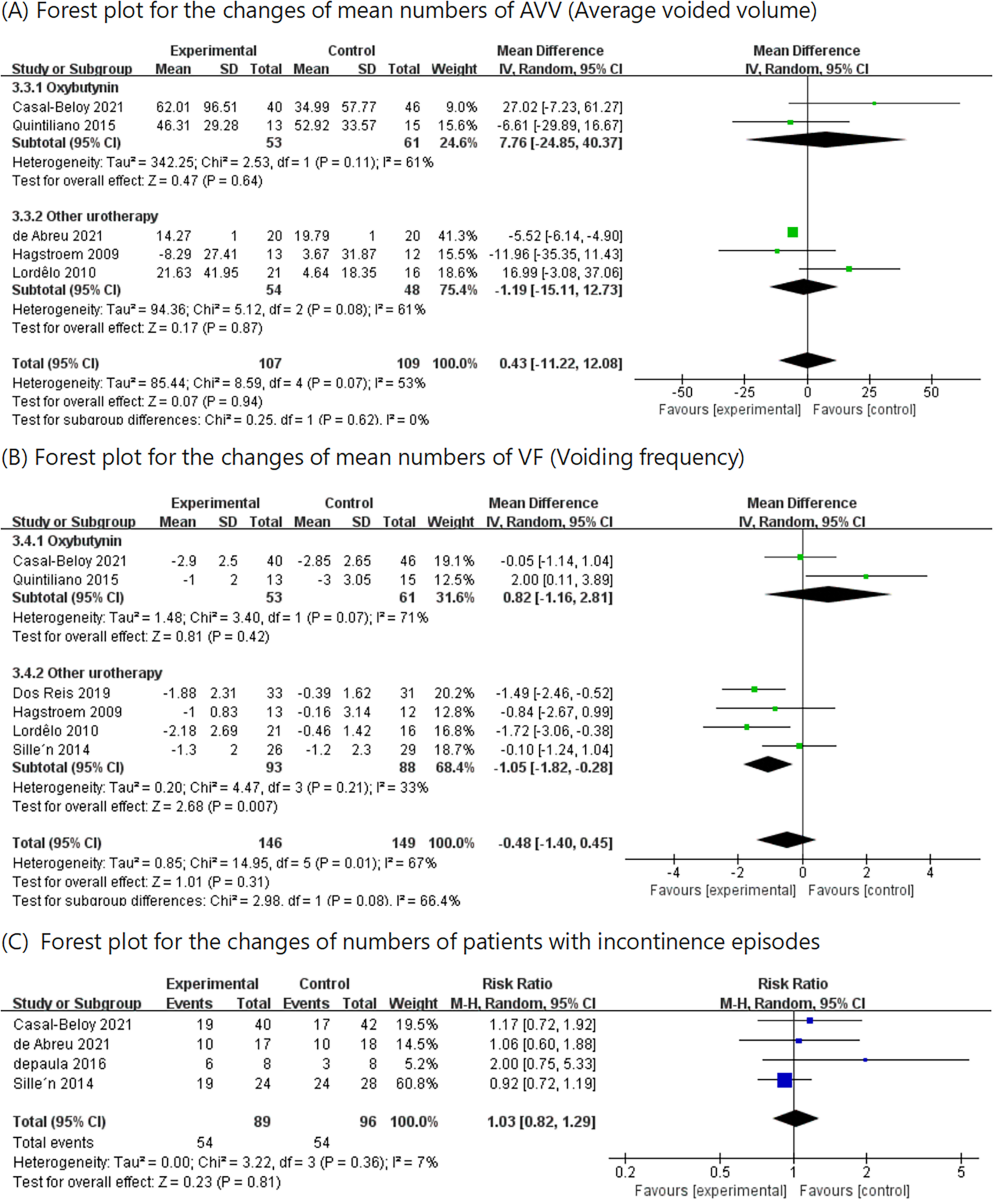
Results of AVV, VF, and change in number of patients with incontinence episodes between the PTENS group and the control group.

Five RCTs (10, 12–14, 17) including 216 patients (107 in the PTENS group and 109 in the control group) were included in this analysis. Comparing the patients receiving oxybutynin treatment and other urotherapy separately, the AVV in the PTENS group showed no significant difference (MD 7.76, 95% CI −24.85 to 40.37; MD −1.19, 95% CI −15.11 to 12.73). There were no statistically significant differences between the PTENS group and the control group (MD 0.43, 95% CI −11.22 to 12.08) (Figure 5).

### 3.5 The changes in mean numbers of VF

In six RCTs (10–15), 295 patients (146 in the PTENS group and 149 in the control group) were included in the analysis. Comparing the patients receiving other urotherapy, there was a significant improvement in voiding frequency in the PTENS group (MD −1.05, 95% CI −1.82 to 0.28). When comparing the patients in the oxybutynin subgroup (MD 0.82, 95% CI −1.16 to 2.81) and those in the total group (MD −0.48, 95% CI −1.40 to 0.45), there was no significant difference (Figure 5).

### 3.6 The changes in the number of patients with incontinence episodes

Four RCTS (10, 11, 16, 17) involving 185 patients (89 in the PTENS group and 96 in the control group) reported data on incontinence episodes (RR 1.03, 95% CI 0.82–1.29; *p* = 0.36), showing that there were no statistically significant differences between the PTENS group and the control group (Figure 5).

### 3.7 The reduction in the number of children with constipation

**Figure 6:**
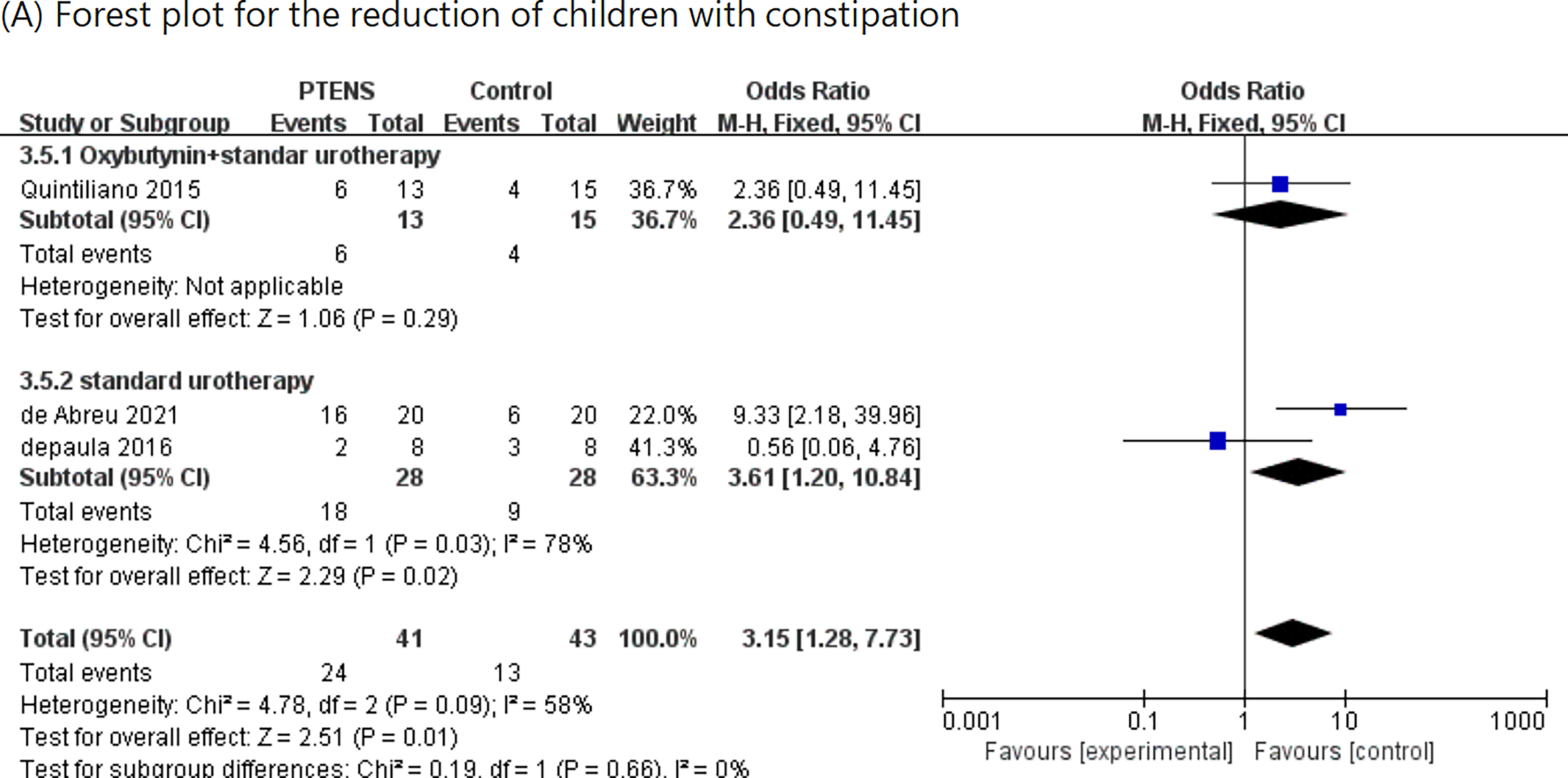
Forest plot for the reduction in the number of children with constipation.

Three RCTs (11, 16, 17) involving 84 patients (41 in the PTENS group and 43 in the control group) reported data on constipation [odds ratio (OR) 3.15, 95% CI 1.28–7.73; *p* = 0.01], showing that the PTENS group had a better effect on constipation when compared with the control group (Figure 6).

## 4 Discussion

Symptoms of overactive bladder, especially UI, significantly impact children's psychological health and physical wellbeing (21). PTENS as an emerging treatment modality was first applied to children with OAB and UI approximately 20 years ago by Hoebeke et al. (6) and Bower et al. (22), with a definitively cured rate of 51.2% and 73.3%, respectively. Even though the mechanism of action still needs to be clarified, most studies have demonstrated good results with this intervention. In a document published in 2017, the ICCS advocated that PTENS could serve as an alternative to standard urotherapy and pharmacotherapy, or be used in children unable to tolerate the side effects of the pharmacotherapy (1, 5).

In this article, we involved eight RCTs and conducted a meta-analysis on bladder function using MVV, AVV, VF, and DVSS, as well as incontinence episodes and constipation. The quality of each RCT was assessed as high. Although the therapeutic benefits and tolerance of PTENS are widely acknowledged in clinical practice, many studies exhibit disparities in methodology. Given the scarcity of studies directly comparing different treatment modalities, we opted to aggregate traditional treatment approaches into a single comparator group for analysis against PTENS. Although this method of aggregation may introduce heterogeneity, the results indicate that it does not obscure the advantages of PTENS.

Among the eight included RCTs, two (14, 17) used placebo/sham stimulation as the control performance, two (10, 12) administered anticholinergic pharmacotherapy, specifically oxybutynin, one study (13) employed electrotherapy at different locations (superficial scapular), and another administered biofeedback. All the control groups performed standard urotherapy except for the studies by Casal-Beloy et al. (10) and Hagstroem et al. (14). Consequently, by performing subgroup analyses, we were able to separate the different methodologies and decrease heterogeneity. Notably, the use of electromyography (EMG) surface electrodes on pelvic floor muscles in one trial may cause controversy. Considering that EMG electrodes do not actively deliver stimulation, we determined this study to be eligible for inclusion in our meta-analysis.

The included studies used diverse outcome measures. By narrowing the inclusion criteria to RCTs, we aimed to obtain more precise results regarding the cause-and-effect relationship between PTENS treatment and OAB syndrome. Furthermore, all RCTs showed consistency in demographics, inclusion criteria, and measured outcomes, thereby enhancing the comparability and generalizability of this meta-analysis.

The stimulation frequency used was 10 Hz, with durations in the range of 20 min–2 h. The time periods ranged from daily to weekly. Most included studies reported favorable outcome for PTENS (10–13, 15–17). However, the treatment protocols across different studies were not entirely consistent, leading to heterogeneity in the analysis. The treatment protocols that were similar and which delivered neuromodulation could be adjusted according to the children's sensitivity threshold, with a generated pulse width of 700 μs and a frequency of 10 Hz (12, 13, 15–17). Five of these studies (12, 13, 15–17) administered PTENS one to three times weekly in sessions of 20 min, while others varied in frequency and duration. Treatment durations were in the range of 1–6 months. Two studies (10, 14) used a different treatment protocol with a lower pulse width of 200 μs. One of them had longer sessions lasting 2 h daily for 4 weeks (14), while the duration of treatment for the other study was 6 months. The study with the shorter duration did not favor PTENS treatment (14).

Based on the successful studies included, the proposed treatment protocol for PTENS would be adjusted according to individual tolerance threshold for current intensity with a generated pulse width of 700 μs and a frequency of 10 Hz. Sessions should be performed for 20 min three times weekly. However, there is currently no consensus on the optimal duration of treatment, indicating the need for further research to define accurate treatment targets in the future.

The success of treatments reported varied significantly across the included studies, reflecting considerable heterogeneity. The criteria outlined by the ICCS (1) were used to define three potential outcomes after the treatment of OAB: “no response” as a <50% reduction in symptoms, “response” as a 50%–99% reduction, and “complete response” as a 100% reduction. Five studies (10, 11, 14, 15, 17) followed the criteria while others used unique outcome measures, such as a visual analog scale (VAS) in the range of 0–10, where 0 indicated no improvement and 10 indicated complete resolution of symptoms. Thus, we performed a meta-analysis for “complete response” only, as detailed in Table 2. The results showed that the PTENS had a better response rate than oxybutynin and other treatments. In terms of the bladder function evaluated by DVSS and other indicators, PTENS showed no significant differences when compared with oxybutynin and other treatments. Regarding adverse effects like constipation episodes, PTENS showed a better effect than standard urotherapy, but had no significant differences when compared with oxybutynin. When it came to the change in the number of patients with incontinence episodes, there were no statistically significant differences.

Anticholinergics are the first-line treatment for OAB. Oxybutynin, in particular, has been used in the treatment of OAB for more than 50 years (23). It competitively suppresses muscarinic receptor-mediated detrusor muscle contractions and exerts direct antispasmodic activity on smooth muscle (23). Despite being orally effective, anticholinergics can lead to side effects such as constipation, dry mouth, and central nervous system (CNS) effects, which limit patient compliance and result in a high discontinuation rate (24).

One of the most common adverse effects in the medication is constipation. Five studies (10, 11, 15–17) mention constipation, but only four studies (10, 11, 16, 17) present the outcome data for constipation. de Abreu et al. evaluated constipation using the Rome IV criteria, while Casal-Beloy et al., de Paula et al., and Quintiliano et al. used the Rome III criteria. Among these studies, only Casal-Beloy et al. preferred PTENS over oxybutynin treatment. Two studies (15, 16) found both treatments to be effective with no significant differences, while the other two did not report outcomes regarding side effects. In addition, one study (11) excluded children with constipation or required them to be treated before inclusion. Other adverse effects of oxybutynin have been reported, such as abdominal pain, dry mouth, increased post-void residual, hyperthermia, and hyperemia (10, 12). Further, anticholinergics may negatively affect colonic transit, exacerbating functional constipation (17). No patients dropped out of these studies due to adverse effects. In contrast, PTENS therapy has no side effects and does not need to be interrupted due to adverse effects, such as dermatitis in the sacral region where the electrodes are applied. To summarize, PTENS is an effective and well-tolerated treatment for children with OAB, with few side effects (25). For children with constipation, PTENS may even improve symptoms (26).

While our meta-analysis results favor PTENS treatment, there are still some limitations that need to be clarified. Positive studies are more likely to be published than negative ones, potentially influencing our analysis due to publication bias. At the same time, most of our research data relied on parent reporting under clinician instruction, introducing subjective uncertainty. In addition, the variety of outcome measures posed challenges with data extraction. Furthermore, due to the relative scarcity of included literature, the results of the analysis may not fully reflect clinical observations.

Therefore, these biases could impact the reliability of the derived results, highlighting the importance of integrating final treatment decisions with individual clinical contexts. In addition, standardizing OAB treatment endpoints may improve the assessment of therapeutic outcomes across various treatment protocols.

## 5 Conclusions

In summary, PTENS has shown better response rates and fewer side effects compared to conventional first-line treatments such as standard urotherapy and antimuscarinic drugs. Clinicians should tailor management strategies to individual patient circumstances when treating children with OAB. However, the findings of this study are limited by the small sample size and imperfect outcomes. Future high-quality RCTs are needed to establish the most effective treatment protocol.

## Data Availability

The original contributions presented in the study are included in the article/Supplementary Material, further inquiries can be directed to the corresponding authors.
